# Hemodynamic Characteristics Associated With Paraclinoid Aneurysm Recurrence in Patients After Embolization

**DOI:** 10.3389/fneur.2019.00429

**Published:** 2019-04-25

**Authors:** Bin Sheng, Degang Wu, Jinlong Yuan, Shanshui Xu, Zhenbao Li, Jin Dong, Niansheng Lai, Xinggen Fang

**Affiliations:** ^1^Department of Neurosurgery, The First Affiliated Hospital of Wannan Medical College, Wuhu, China; ^2^Department of Nursing, The First Affiliated Hospital of Wannan Medical College, Wuhu, China

**Keywords:** hemodynamic, aneurysm, embolization, recurrence, computational fluid dynamic

## Abstract

**Objective:** To investigate the hemodynamic features before and after embolization of paraclinoidal aneurysms using hemodynamic numerical simulation and the influence of embolization on recurrence after embolization.

**Methods:** From January 2016 to December 2017, we enrolled a total of 113 paraclinoidal aneurysms treated with embolization. They were divided into recurrent group and stable group depending on follow-up results. An aneurysm model was generated based on 3D-DSA before and after embolization. The hemodynamic characteristics were analyzed between two groups using Computational fluid dynamic (CFD).

**Results:** In the recurrent group, the peak systolic WSS, OSI and velocity around the aneurysm neck areas prior to embolization were 20.47 ± 3.04 Pa, 0.06 ± 0.02 and 0.07 ± 0.03 m/s, respectively. These values were 23.50 ± 4.11 Pa, 0.06 ± 0.01 and 0.11 ± 0.02 m/s, respectively in the stable group (*P* > 0.05). The WSS, OSI, velocity around the same areas in the recurrent group after embolization were 35.59 ± 8.75 Pa, 0.07 ± 0.02 and 0.12 ± 0.03 m/s, respectively (*P* < 0.01). In the stable group, the WSS, OSI and velocity were 13.08 ± 2.89 Pa, 0.04 ± 0.01 and 0.07 ± 0.02 m/s, respectively (*P* < 0.01). After embolization, the WSS, OSI and velocity around the aneurysm neck areas in the recurrent group were significantly higher than those in the stable group.

**Conclusions:** High peak systolic WSS, OSI and velocity around aneurysm neck areas after embolization of paraclinoidal aneurysms may be important factors leading to recurrence.

## Introduction

Paraclinoid aneurysms are defined as aneurysms arising from the segment of the internal carotid artery (ICA) between the distal dural ring and the origin of the posterior communicating artery. They account for approximately 1.3–5% of all intracranial aneurysms and they comprise the majority of such aneurysms in females (1–3). Hemodynamic characteristics are thought to be the most important risk factors for occurrence, growth and rupture of intracranial aneurysms (4, 5). Because of the complex anatomy and the inherent risks associated with microsurgery as well as technological advancements in vascular imaging, endovascular techniques have been used increasingly to treat paraclinoid aneurysms. Though endovascular therapy reduces the difficulty of treatment, the postoperative recurrence rate of 2.7–17.8% remains a difficult problem that is too important to neglect in clinical practice (6–8). The size of the aneurysm dome, occurrence of rupture, Hunt-Hess grade, density of embolization, presence of stents and changes of hemodynamics often affect long-term stability after aneurysm embolization. Computational fluid dynamic (CFD), as a relatively new discipline that employs computer technology to simulate and analyze hydrodynamics, has been used to explore the effects of hemodynamic characteristics on aneurysms. Studies (3, 9, 10) have confirmed that the hemodynamic changes in aneurysms play important roles in the development of aneurysms, high wall shear stress and high velocity in the blood flow impact field may promote the aneurysmal growth. The present study was undertaken to analyze these hemodynamic characteristics in paraclinoid aneurysms and to investigate the role of those characteristics in recurrence after aneurysm embolization.

## Materials and Methods

Study participants were recruited from the Department of Neurosurgery, the First Affiliated Hospital of Wannan Medical College, Wuhu City, China. The study was performed in accordance with the Declaration of Helsinki and all participants provided written informed consent. The study was approved by the Research Ethics Committee of Wannan Medical College.

### Study Design

From January 2016 to December 2017, we enrolled 109 patients with 113 paraclinoid aneurysms who underwent endovascular treatment and met inclusion criteria. Multiple aneurysms were calculated separately per aneurysm. Inclusion criteria were as follows: (1) diagnosis of paraclinoid aneurysm by angiography; (2) complete clinical history data; (3) three-dimensional (3D) digital subtraction angiography (DSA) images of the paraclinoid aneurysm adequate for CFD analysis; and (4) informed consent of patient or family. Exclusion criteria were as follows: (1) no paraclinoid aneurysm; (2) lack of completed clinical history data or loss to follow-up; (3) non -endovascular treatment; and (4) DSA images of the paraclinoid aneurysm not adequate for CFD analysis.

### Modeling of the Aneurysms and Numeric Simulation

Patient-specific 3D-DSA data for all paraclinoid aneurysms were obtained prior to treatment, then hemodynamic analysis was carried out using a finite element method. Three-dimensional vascular images of intracranial aneurysms were obtained on a GE Medical Systems LCV and a three-dimensional angiography device. We removed the unrelated branches of small vessels and transferred data to a systematic workstation. Data were then imported into self-programmed MATLAB 7.0 (MathWorks, Natick, MA, US) to calculate and synthesize three-dimensional images in STL format. The grids were divided by the software ICEM CFD16.0 (ANSYS Inc, Canonsburg, Pennsylvania, US) where STL files were imported for analysis of hemodynamic changes. In order to improve computational accuracy and computational efficiency, the grids were divided into 1.3 million to 2 million segments (11). After the grids were divided, CFX16.0 (ANSYS Inc., USA) software was used for hemodynamics analysis. Each cardiac cycle was divided into 800 steps, each with a duration of 0.001 s.

The software of CFX 16.0 was used to solve the flow-governing Navier-Stokes equations with the assumption of laminar and incompressible flow. The blood vessel wall was assumed to be rigid with no-slip boundary conditions and Newtonian blood flow. The density and dynamic viscosity of the blood were set to 1.05 × 10^3^ kg/m^3^ and 0.00345 Ns/m^2^, respectively. The pulsatile velocity profile obtained by transcranial Doppler from a normal subject was applied for the inflow boundary conditions. We performed three cardiac cycle simulations for numerical stability and took the last cardiac cycle as output.

The inlet condition of the vessel was set to the pulsatile velocity obtained by transcranial Doppler, and the outlet condition was set as the stress-free condition. The initial blood pressure and flow velocity were set to 0. The hemodynamic parameters of paraclinoid aneurysms were consistent in each cardiac cycle, but the changes in the number were due to the fluctuation of blood pressure. The peak of velocity and WSS appear at the end of systolic blood pressure, so we merely compared the hemodynamic characteristics of the systolic period of the heart (*T* = 0.2 s).

### Statistical Analysis

All data were analyzed using SPSS Statistic version 20.0 (SPSS, Inc., Chicago, Illinois, USA). Data were presented as mean ± SD. Comparisons were performed using the unpaired Student *t*-test for normal variation variables. The Mann-Whitney U test was used to assess the differences between the groups. Paired *t*-tests were used in the preoperative and postoperative groups in the same group of patients, and the independent sample *t*-test was used for comparisons of various groups of preoperative patients. *P* < 0.05 was considered statistically significant.

## Results

### Characteristics of Patients With Paraclinoid Aneurysm in the Recurrent and Stable Groups

We created 3D reconstructions of 113 paraclinoid aneurysm models. These aneurysms were divided into two groups depending on whether they recurred: recurrent group (*n* = 17) and stable group (*n* = 96). The population consisted of 75 females and 38 males with mean age 53.76 ± 7.73 years in the recurrent group (10 females and 7 males) and 57.15 ± 10.79 years in the stable group (65 females and 31 males). There were 44 patients with hypertension and 28 with diabetes mellitus. There were no significant differences in terms of gender, age, smoking status, hypertension, diabetes or presence of stent (*P* > 0.05). However, there were significant differences in terms of embolization density and occurrence of ruptured aneurysm (*P* < 0.05). These data are shown in Tables 1, 2.

**Table 1:** Clinicopathologic features of 113 paraclinoid aneurysms between the recurrent and stable groups.

**Clinicopathologic parameters**	**Recurrent group(*n* = 17)**	**Stable group(*n* = 96)**	***P*-value**
**GENDER**			
Male	7	31	0.475
Female	10	65	
**SOMKING**			
Yes	6	39	0.679
No	11	57	
**HYPERTENSION**			
Yes	9	35	0.199
No	8	61	
**DIABETES**			
Yes	5	23	0.631
No	12	73	
**STENT ASSISTED**			
Yes	5	43	0.237
No	12	53	
**COMPLETELY OBLITERATION**			
Yes	9	80	0.005
No	8	16	
**RUPTURE**			
Yes	10	14	< 0.001
No	7	82	

**Table 2:** Morphologic parameters of 113 paraclinoid aneurysms between the recurrent and stable groups.

**Parameters**	$\bar{\text{x}}$ **±** **s**		***t***
	**Recurrent group (*n* = 17)**	**Stable group (*n* = 96)**	
Age	53.76 ± 7.73	57.15 ± 10.79	−1.235
Aneurysm size, mm	7.65 ± 2.45	6.44 ± 1.63	2.59
Aneurysm lengh, mm	3.41 ± 0.79	3.16 ± 0.82	1.183
AR	0.45 ± 0.07	0.48 ± 0.07	−1.716
Follow-up interval, month, M(range)	10(7–18)	8(3–12)	18.58^a^

a *: χ2, AR: aspect ratio*.

### Hemodynamic Analysis of Paraclinoid Aneurysms Before Embolization

**Figure 1:**
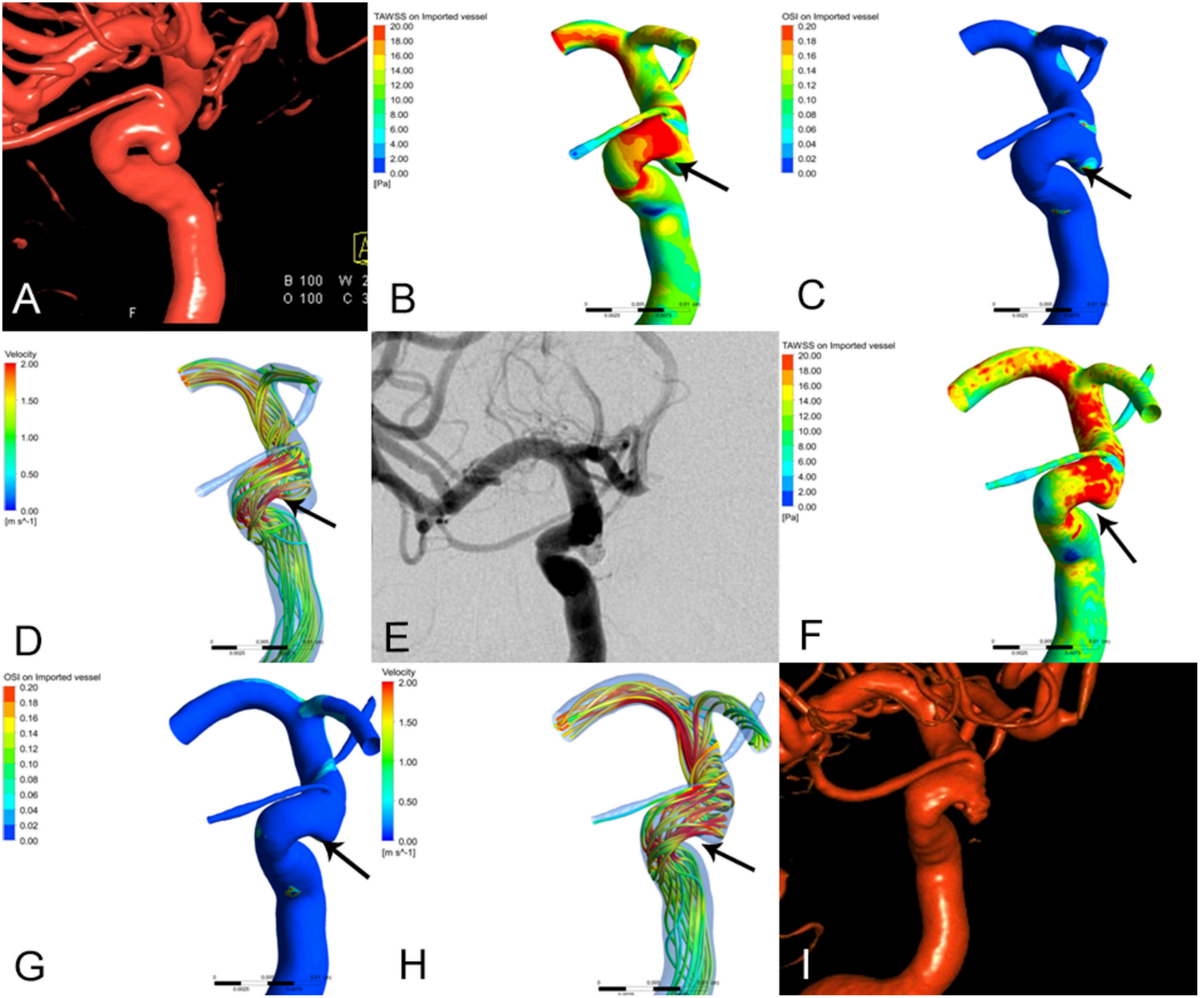
The images of preoperative and postoperative angiographic and hemodynamics simulations in recurrence group of paraclinoid aneurysms. **(A)** 3D-DSA shown left paraclinoid aneurysm prior to embolization. **(B–D)** Changes of hemodynamic characteristics prior to embolization. **(B)** Wall shear stress (WSS), **(C)** oscillatory shear index (OSI), **(D)** blood flow velocity (V). **(E)** DSA shown the aneurysm was completely embolism after embolization. **(F–H)** Changes of hemodynamic characteristics after embolization. **(F)** Wall shear stress (WSS), the peak systolic WSS around the aneurysm neck areas is significantly higher than those prior to embolization (the arrow show, *T* = 0.2 s). **(G)** Oscillatory shear index (OSI), the oscillatory shear index around the aneurysm neck areas is moderately higher than those prior to embolization (the arrow show, *T* = 0.2 s). **(H)** Blood flow velocity (V), the blood flow velocity is more irregular than those prior to embolization, that is, the large regular streamlines in the neck of aneurysm become relatively irregular streamlines. (the arrow show, *T* = 0.2 s). **(I)** 3D-DSA shown that the aneurysm was recrudescent after 6 month embolization.

**Figure 2:**
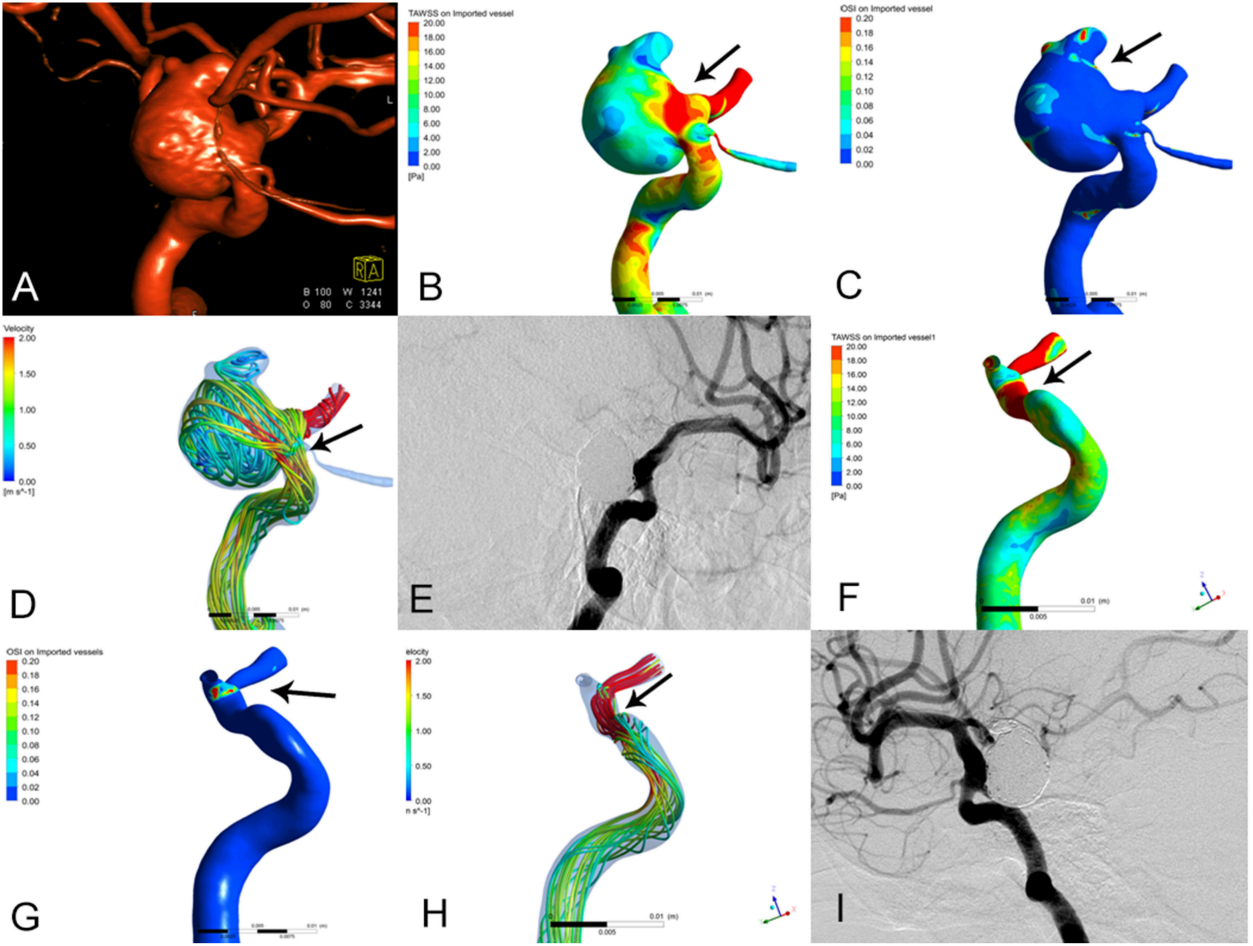
The images of preoperative and postoperative angiographic and hemodynamics simulations in stable group of paraclinoid aneurysms. **(A)** 3D-DSA shown right paraclinoid aneurysm prior to embolization. **(B–D)** Changes of hemodynamic characteristics prior to embolization. **(B)** Wall shear stress (WSS), **(C)** oscillatory shear index (OSI), **(D)** blood flow velocity (V). **(E)** DSA shown the aneurysm was completely embolism after embolization. **(F–H)** Changes of hemodynamic characteristics after embolization. **(F)** WSS, the peak systolic WSS around the aneurysm neck areas is significantly lower than those prior to embolization (the arrow show, *T* = 0.2 s). **(G)** OSI, the oscillatory shear index around the aneurysm neck areas is moderately lower than those prior to embolization (the arrow show, *T* = 0.2 s). **(H)** V, the blood flow velocity is more regular than those prior to embolization, that is, the large irregular streamlines in the neck of aneurysm become relatively regular streamlines. (the arrow show, *T* = 0.2 s). **(I)** DSA shown that the aneurysm was stable after 6 month embolization.

Compared to the numerical simulation of hemodynamics between recurrent and stable groups, the blood flow pattern in the parent artery and paraclinoid aneurysm sac were almost consistent. Blood entered the aneurysmal sac from the impact field of the parent artery to form a complex vortex, and high wall shear stress was observed in the area of the impact field (Figures 1, 2). Peak systolic WSS, OSI and blood velocity were moderate higher in the stable group than in the recurrence group before embolization, these results are shown in Table 3.

**Table 3:** Comparison of peak systolic WSS, OSI, and velocity between two groups of patients before and after embolization.

**Groups**	***n***	**WSS (Pa)**			***t***	**OSI**			***t***	**V (m/s)**			***t***
		**PR**	**PO**	**PR-PO**		**PR**	**PO**	**PR-PO**		**PR**	**PO**	**PR-PO**	
Recurrent group	17	20.47 ± 3.04	35.59 ± 8.75	−15.12 ± 7.20	−8.65^a^	0.06 ± 0.02	0.07 ± 0.02	−0.01 ± 0.01	−7.98^a^	0.07 ± 0.03	0.12 ± 0.03	−0.05 ± 0.03	−6.22^a^
Stable group	96	23.50 ± 4.11	13.08 ± 2.89	10.42 ± 2.99	34.05^a^	0.06 ± 0.01	0.04 ± 0.01	0.02 ± 0.01	17.90^a^	0.11 ± 0.02	0.07 ± 0.02	0.03 ± 0.01	18.62^a^
		2.89	−13.12^a^	14.40^a^		0.017	−7.22^a^	11.78^a^		4.33	−7.45^a^	10.12^a^	

a *p < 0.001, PR, prior to embolization; PO, post embolization; V, blood flow velocity*.

### Hemodynamic Analysis of Paraclinoid Aneurysms After Embolization

The blood flow patterns in the recurrent and stable groups changed apparently after embolization. Compared to the stable group, the peak systolic WSS, OSI and blood flow velocity around the same areas in the recurrent group after embolization were 35.59 ± 8.75 Pa, 0.07 ± 0.02 and 0.12 ± 0.03 m/s, respectively. These values were significantly higher than those before embolization (*P* < 0.01). By contrast, in the stable group, peak systolic WSS, OSI and blood flow velocity were 13.08 ± 2.89 Pa, 0.04 ± 0.01 and 0.07 ± 0.02 m/s, respectively. These values were significantly higher than those before embolization (*P* < 0.01). After embolization, the peak systolic WSS, OSI and blood flow velocity around the aneurysm neck areas in the recurrent group were significantly lower than those in the stable group (Table 3).

## Discussion

Intracranial aneurysms, especial paraclinoid aneurysms, are a significant cause of morbidity (1.3–5%) (2, 3). Though paraclinoid aneurysms have been treated surgically, endovascular treatment has been adopted in recent years (1). With decreasing the difficulty of endovascular treatment with paraclinoid aneurysms, the postoperative recurrence rate had increased as well. The existing literature (4, 5, 12, 13) suggested that the size of the aneurysm dome, occurrence of rupture, presence of stent and especially changes in hemodynamics were associated with long-term postoperative stability. Therefore, an understanding analysis of hemodynamic changes is urgently needed. Computational fluid dynamic (CFD), a powerful simulation tool that quantitatively analyzes the morphology and hemodynamics of intracranial aneurysms, has been used to explore the effects of hemodynamics characteristics on aneurysms. A growing body of literature (14, 15) reported that hemodynamics play an important role in recurrent aneurysms, and many parameters are associated with aneurysm recurrence, including wall shear stress (WSS), oscillatory shear index (OSI), and blood flow velocity. Thus, the purpose of this study was to analyze the hemodynamic characteristics of paraclinoid aneurysms in order to assist physicians in adjusting therapy to reduce the rate of recurrence for patients with paraclinoid aneurysms.

WSS is one of the most thoroughly-evaluated hemodynamic parameters associated with aneurysm recurrence. Ferns et al. (16) reconstructed aneurysm models and analyzed hemodynamic characteristic by computer. They determined that the most important factor was WSS, especially at the parent artery, where the value is three time higher than the WSS at the aneurysm sac. Cebral et al. (17) reported that the growth of the aneurysm sac occurs in regions of higher WSS. Other studies (9, 18) reported that high WSS was closely related to formation of aneurysms. By contrast, the regeneration of aneurysms was associated with lower WSS. Ortega et al. (19) used computer technology to artificially remove the aneurysm sac at the end of basilar artery and simulated hemodynamic changes after embolization. They observed a new impact domain and high WSS region at the local residual neck. They concluded that the high WSS region may be related to changes of blood flow morphology in the aneurysm sac after embolization. Francis et al. (20, 21) reported that high WSS induced apoptosis of vascular endothelial cells after pathological processes such as deposition of low-density lipoprotein, destruction of the vascular intima and thinning of vascular stroma, this led to the reconstruction of vascular wall. These may be important mechanisms for the growth of recurrence of aneurysms after embolization. Luo et al. (22, 23) compared the hemodynamic parameters of 17 patients with totally occluded intracranial aneurysms and demonstrated that the WSS in the region of near complete or complete embolization of aneurysms was higher than that of other regions, possibly playing an indispensable role in the recurrence of aneurysms. The present study also showed that the WSS was significantly higher compared to those before embolization in the recurrence group. By contrast, in the stable group, the WSS was significantly lower than those before embolization. These results are consistent with those of previous reports.

OSI is a dimensionless parameter used to measure the change in direction of WSS during the cardiac cycle. It is another important hemodynamic parameter associated with the recurrence of aneurysms. Jeong et al. (24) studied hemodynamic alterations caused by vascular angular deformation in stent-assisted coiling of bifurcation aneurysms; they found that OSI was related to the recurrence of aneurysms and the highest OSI was found near the distal neck of non-stent daughter branch. Isoda et al. (25) analyzed hemodynamics by magnetic resonance fluid dynamics based on time-resolved three-dimensional phase-contrast MRI; they found that the OSI at the apex of spiral flow was higher than that of the whole aneurysmal wall. This may lead to the recurrence of aneurysms. Machi et al. (26) analyzed six small cerebral aneurysms to differentiate focal vs. general growth and to analyze the hemodynamic environment. They found the aneurysmal bleb formation was associated with higher OSI. These studies drew similar conclusions to the effect that recurrence of aneurysms after embolization is closely related to high OSI. These results accord with those of our study.

Blood flow velocity is a similar parameter associated with the recurrence of aneurysms. Faster blood flow correlates with greater the impact of local blood vessels. It is easy to cause thinning of local blood vessel wall following long-time impact. Chatziprodromou and Rayz et al. (27, 28) reported that high WSS and accompanying high blood flow velocity were not conducive to thrombosis in the aneurysm cavity. Intimal hyperplasia in the region of aneurysm neck and thrombosis formation in the aneurysm cavity play important roles in long-term stability after embolization. Moreover, high blood flow velocity at the remnant of the neck has an impact on the coiling in the aneurysm cavity that can compress the coiling. This is another factor that causes the recurrence of aneurysms after embolization (29). In the present study, high blood flow velocity in the recurrence group is consistent with the results of these studies.

There are some potential limitations in our study. First, in the process of establishing paraclinoidal aneurysm models, the small vessel branches around the parent artery were artificially removed. Doing so may lead to inaccurate results and less accurate conclusions. Second, this was a single-center study with a small sample size. Further studies with larger populations are needed to confirm our results. Third, in addition to hemodynamic parameters, morphological variables are also important factors influencing the recurrence of aneurysm after embolization. These variables include size of aneurysm dome, aspect ratio, size ratio, dome-to-neck ratio, bottle neck ratio and inflow angle. Some of these parameters, especially embolization density and presence of ruptured aneurysm, have been confirmed to play important roles in the recurrence of aneurysms.

Another limitation in our study is that we cannot accurately simulate the real hemodynamic data of stent-assisted coiled or Flow Diversion in the treatment of aneurysm. Tremmel et al. (30) reported about the aneurysmal hemodynamics by stent-assisted that aneurysmal WSS was significantly reduce with stent-assisted embolization, and it was decreased with increasing number of deployed stents. It conclude that using stent increase the likelihood of inducing aneurysm thrombotic occlusion. Liu et al. (31) studied the effect of hemodynamics on 27 paraclinoid aneurysms after stent-assisted coil embolization and demonstrated that aneurysm flow velocity significantly decreased by stent placement. These study showed that stent-assisted coiled or Flow Diversion may decrease the recurrence of aneurysm. With the software of CFD improving, we believe that we can draw an accurate conclusion. Finally, because of the complexity and time-consuming process of CFD research, the protocols need to be simplified to further improve the simplicity and effectiveness of the process. Finally, because of the complexity and time-consuming process of CFD research, the protocols need to be simplified to further improve the simplicity and effectiveness of the process.

## Conclusions

Peak systolic WSS around aneurysm neck areas is always higher than in other regions. After embolization, high peak systolic WSS, high OSI and high blood flow velocity around the aneurysm neck areas are associated with aneurysm recurrence.

## Ethics Statement

The Research Ethics Committee of Wannan Medical College.

## Author Contributions

BS, SX, and JY contributed the conception, design, data analysis. NL, ZL, and JD collected the data and drafted the manuscript. DW and XF conception and design, final approval of the version to be published.

### Conflict of Interest Statement

The authors declare that the research was conducted in the absence of any commercial or financial relationships that could be construed as a potential conflict of interest.
